# Infesting Seaweeds as a Novel Functional Food: Analysis of Nutrients, Antioxidants and ACE Inhibitory Effects

**DOI:** 10.3390/ijms25147588

**Published:** 2024-07-10

**Authors:** Annalaura Brai, Anjeza Hasanaj, Chiara Vagaggini, Federica Poggialini, Elena Dreassi

**Affiliations:** Department of Biotechnology, Chemistry and Pharmacy, University of Siena, via A. Moro, 53100 Siena, Italy; anjeza.hasanaj@student.unisi.it (A.H.); chiara.vagaggini@student.unisi.it (C.V.); federic.poggialini@unisi.it (F.P.)

**Keywords:** seaweeds, antioxidants, proteins, essential amino acids, ACE, cardiovascular diseases

## Abstract

Globalization and climate change are both contributing to an increase in the number of potentially invasive algae in coastal areas. In terms of biodiversity and financial losses, the invasiveness of algae has become a significant issue in Orbetello Lagoon. Indeed, studies from the Tuscany Regional Agency for Environmental Protection show that the reduction in dissolved oxygen caused by algal diffusion is detrimental to fisheries and biodiversity. Considering that wakame and numerous other potentially invasive seaweeds are consumed as food in Asia, we assess the nutritional and nutraceutical qualities of two potentially invasive seaweeds: *Valonia aegagrophila* and *Chaetomorpha linum*. We found that both algae are a valuable source of proteins and essential amino acids. Even if the fat content accounts for less than 2% of the dried weight, its quality is high, due to the presence of unsaturated fatty acids. Both algae are rich in antioxidants pigments and polyphenols, which can be exploited as nutraceuticals. Most importantly, human gastrointestinal digestion increased the quantity of polyphenols and originated secondary metabolites with ACE inhibitory activity. Taken together, our data strongly promote the use of *Valonia aegagrophila* and *Chaetomorpha linum* as functional foods, with possible application in the treatment of hypertension and cardiovascular diseases.

## 1. Introduction

The Food and Agriculture Organization (FAO) estimates food production must rise by 60% to fulfil the demands of the world’s expanding population, which is estimated to reach over 9 billion people in 2050 [1,2]. In parallel, the Institutions are discouraging the intensive farming of animal sources like cattle, due to the necessity to reduce the emissions of CH_4_, CO_2_, and N_2_O pollutants. In this context, seas and oceans represent important but poorly exploited sources of food and feed. Despite accounting for more than 70% of the Earth’s surface, only 2% of human food comes from the seas and the oceans [3]. Accordingly, the European Green Deal, the Farm to Fork Strategy and the Sustainable Blue Economy Communication strongly promote the use of algae as a source of food and feed, and recommend novel economic strategies to increase the farming of both seaweed and microalgae [4,5]. This strategy has multiple advantages. On the one hand, algae have a low carbon footprint; on the other hand, algae are a food rich in fibers, proteins and nutraceutical compounds [6]. There are between 25 and 30 thousand species of algae, ranging widely in size and form from unicellular microscopic algae called microalgae to large multicellular organisms called seaweeds (SWs). SWs are categorized into three major groups determined by considering the pigment that makes up the group and the mechanism by which it uses photosynthesis: the Rhodophytas, or red algae; the Chlorophytas, or green algae, which are both parts of the Plantae kingdom; the Ochrophytas, or brown seaweed [7]. Some algae can live in extreme conditions, thanks to their ability to quickly adapt to new environmental conditions to survive including extreme temperature, variations in salinity, UV–vis irradiation and nutrients. As a result, they produce a wide range of secondary biologically active metabolites that are unique to their species [8]. In addition, algae are also valued for their ease of cultivation, quick growth and the ability to regulate the production of bioactive components through the modification of cultivation conditions. According to market analyses [9,10], the demand for seaweed may increase up to 9 billion in 2030, due to the health benefits associated and to the growing numbers of vegetarians and vegans. Even if algae are an essential part of the diet in many Asian nations, their diffusion in Europe is in many cases limited to few species like the most famous nori (*Porphyra* species) and spirulina (*Arthrospira platensis).* After the recent inclusion of more than 20 algae in the EU novel food catalogue, the marketable algae have reached more than 60 entries, expanding the list of available products beyond the most famous classical nori that surrounds sushi rolls [11]. Kombu (*Saccharina japonica*), is a well-known brown seaweed, rich in fibers, microelements, vitamins and omega-3. It is adopted for the preparation of salads or sushi.

The number of algae with invasive potential is increasing in coastal locations across the globe as a result of both climate change and growing globalization. Other factors can be taken into account, in particular the development of aquaculture, fisheries and marine tourism [12]. In addition to displacing other native species, reducing biodiversity, altering the composition and function of the native ecosystem, these algae also result in losses for the fishing and numerous other industrial sectors that are connected to the aquatic environment. Nevertheless, the abundance of SWs can represent a resource and it is fundamental to investigate potential uses to enhance the value of infesting SWs.

Wakame (*Undaria pinnatifida*) is another popular alga, used in preparation of the miso soup. A brown seaweed, wakame is so easy to farm in temperate climates that it has been included in the list of the invasive species, which can compete and reduce the presence of native algal and fauna species [13]. In Asia, wakame is considered an important nutraceutical food, rich in antioxidants, such as fucoxanthin and omega-3 fatty acids or is used in traditional medicine. Considering the example of wakame, and many other invasive SWs that are used in Asia as a food, we decided to evaluate the nutrient composition and the nutraceutical properties of two seaweeds that are potentially invasive, *Valonia aegagrophila* (VA) and *Chaetomorpha linum* (CL). Since 1999, the eutrophication caused the diffusion of novel seaweeds in the Orbetello lagoon (Italian West Coast). In recent years, the invasiveness of these algae has represented an important problem in terms of biodiversity and economic losses. In fact, according to the data recorded by the Tuscany Regional Agency for Environmental Protection (ARPAT), the algal diffusion is causing the reduction in dissolved oxygen, detrimental to biodiversity and for fish industry [14]. The local Institutions have made many efforts to contain the problem, including pumping water from the sea, confining wastewater, and mechanically removing the macroalgae [15]. The harvested macroalgae, represent an expensive biowaste, accounting for more than 6 tons per year. According to circular economy, additional efforts have been made to upcycle this residue into cosmetical or pharmaceutical products, or to produce biodiesel [16,17,18]. Even if CL and VA are not yet included in the list of EFSA-approved edible algae, they are an important ingredient in Asia traditional dishes such as soups or lawar [19]. Other ethnical uses include the preparation of cosmetics or the use as pharmaceuticals. The use of CL and VA as traditional food, reassures about the possible toxic effects of the two SWs. In addition both SWs are members of green algae, characterized by high nutrient content, in particular essential amino acids and long-chain polyunsaturated fatty acids [20,21]. Noteworthy, green algae are rich in phlorotannins, a source of polyphenolic compounds, which coupled with lipophilic pigments and secondary metabolites make green SWs a valuable source of antioxidants with many cosmetic and pharmaceutical applications [22].

## 2. Results

### 2.1. Determination of Antioxidant Compounds and Antioxidant Potential of Seaweeds

The dried algae have been analyzed for their content of residual water (Table 1). The residual water remained below 10% for VA, CL and SJ, while the commercial PP showed a residual quantity of 12%. The nutrient composition is influenced by the territory, seasonal changes and harvesting period [23]. Fats have been determined using the Folch method, showing negligible levels below 2% for all the seaweeds (Table 1), as previously reported [24]. The total protein content has been determined with the Kjeldahl method, using a conversion factor of 6.25. The protein content ranges from 8.1 to 30.7% (Table 1). Even in this case, the protein content is related to the harvesting period, accordingly, we found values comparable to those already reported for May [25,26,27]. As for the other nutrients, carbohydrates vary during the year. Considering that, as previously reported carbohydrates in green SWs can vary from 26% to 40% of the DW [25,28], and 100 g of dried seaweeds can furnish from 240 to 350 kcal. Seasonal changes seem to be particularly important for nutrient composition [23].

Amino acidic composition has been determined after acidic hydrolysis. As reported in Table 2, the most abundant amino acids are glutamic and aspartic acid which contribute to the characteristic umami taste of SWs [29]. Even if no data regarding the amino acidic composition of CL and VA are available, data are in agreement with those reported for commercial SWs [29,30], which, as highlighted for main nutrients, are influenced during the year. SWs contain all the essential amino acids, in particular threonine, lysine and isoleucine, comparison with ONU/FAO/WHO requirements are reported in Table S1.

Antioxidant compounds are important to prevent cardiovascular diseases and cancer. With the purpose of better understanding the antioxidant potential of infesting seaweeds, the main antioxidant compounds were analyzed. As reported in Table 3, the content of polyphenols ranges from 1.03 to 5.82 mg of gallic acid equivalent per gram of dry weight (GA/g DW). The flavonoid content ranges from 1.02 to 29.67 mg of quercetin equivalent (QE)/g DW. The quantity of antioxidant compounds was significantly higher in PP. The total antioxidant activity (TAC) was thus evaluated using ABTS and DPPH assays. As reported in Table 4 the highest values were found in PP, followed by VA, CL and SJ. Interestingly, ABTS data correlated with flavonoid and proanthocyanidin content and DPPH data, on the contrary, no correlations were found between DPPH and TPC, and DPPH and proanthocyanidins. Data are comparable to those reported by Farvin et al. [31], but higher than those reported by Stabili et al. [21].

### 2.2. Analysis of the Lipidic Composition of Seaweeds

**Figure 1:**
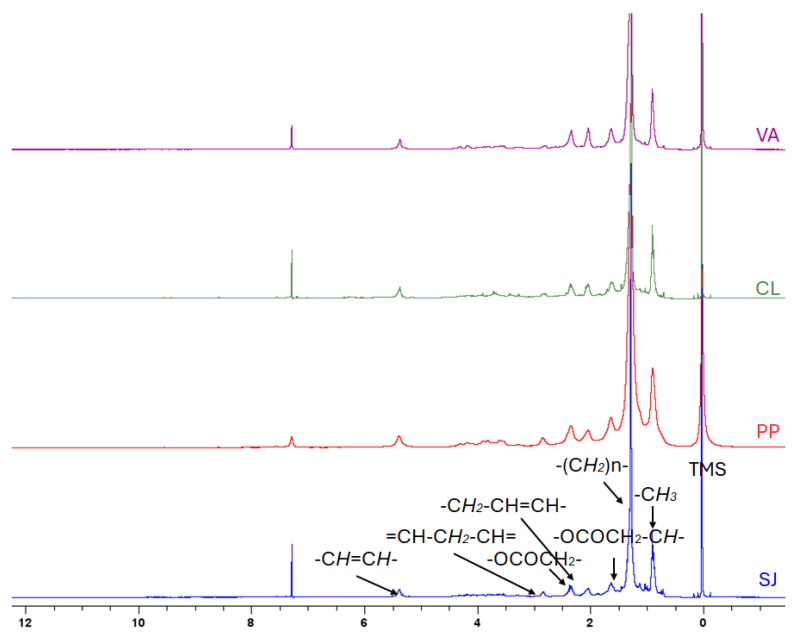
^1^HNMR of the lipophilic extracts. *Valonia aegagrophila* (VA) (violet), *Chaetomorpha Linum* (CL) (green), Nori (Porphyra purpurea, PP) (red) and Kombu (*Saccharina japonica* SJ) (blue).

The lipophilic extracts were used to determine the amount of fats and the fat composition. As reported in Table 1, the fat quantity was limited and ranged from 1.09 to 1.46%. The fat extracts were analyzed by ^1^HNMR and GC. NMR signals were assigned by comparison with the published data [32,33] and Chenomx NMR suite v 11 (Chenomx Inc., Edmonton, AB, Canada). As reported in Figure 1, the main components of lipidic extracts are represented by saturated and unsaturated fatty acids sterols and triacylglycerols. The magnification of the region between 11.5 and 8.5 ppm (Figure S1A, Supplementary Information) highlights the presence of chlorophylls, while carotenoids and xanthophylls signals were found in the regions between 6.8 and 6.5 ppm (Figure S1B, Supplementary Information).

In order to further analyze the metabolites observed, GC-MS analysis has been performed, followed by quantification of the compounds by GC equipped with a flame ionization detector (FID). As reported in Table 5, seaweeds are rich in MUFAs and PUFAs, with quantities ranging from 19% to 39% for MUFAs and from 16% to 27% for PUFAs. Among the SFAs, palmitic acid was the most abundant in all the SWs analyzed, palmitoleic and vaccenic acid among MUFAs and hexadecatrienoic acid among PUFAs. SFAs were significantly lower in SJ, and increased in PP, CL and VA. In contrast, SJ was particularly rich in MUFAs, followed by CL, VA and PP. Among the PUFAs SJ and PP have statistically comparable results, followed by VA and CL. The n6/n3 ratio was below 10 in all the SWs, except PP, with a ratio of 16. VA and CL have AI comparable to PP, in accordance with data already published for green SWs [20,23].

Chlorophylls, carotenoids and xanthophylls were finally quantified, using a spectrophotometric method [34]. As reported in Table 6, seaweeds were rich in chlorophylls and pheophytins, with concentrations ranging from 27.39 µg to 4476 µg per gram of dry weight. The carotenoids content ranges from 3.8 to 61 µg per gram of dry weight, slightly below the values already reported using polar solvents like methanol [32,35]. Notably, CL extracts as preliminarily observed in NMR spectra, have the highest concentration of pigments.

### 2.3. Analysis of Gastrointestinal Digestion Effects on Polyphenol Secondary Metabolites Composition and ACE Inhibitory Activity

**Figure 2:**
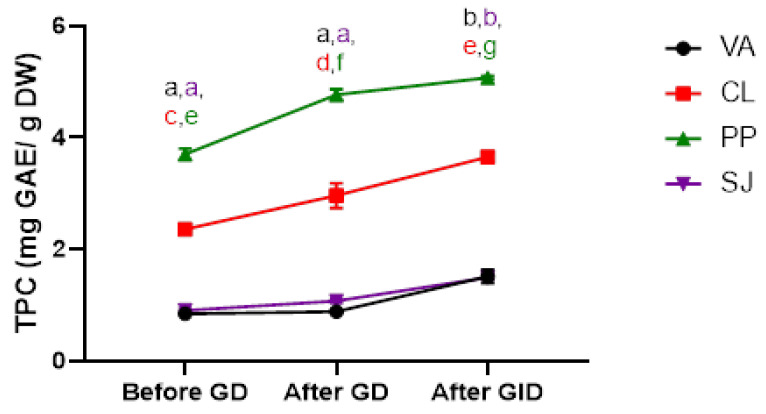
Total phenol content (TPC) before and after gastric digestion (GD) and gastrointestinal digestion (GID). Values are expressed as the mean ± SD. ^a–g^ Different superscript letters indicate a significant difference among the means.

The bioaccessibility of polyphenols is often limited and can be influenced by the stability of compounds and complex interactions in the food matrix. We simulated in vitro the gastrointestinal digestion of seaweeds, by incubating the algal bolus with pepsin at pH 2 for 2 h to simulate gastric fluids, followed by treatment with trypsin and chymotrypsin at pH 6.5 to simulate duodenal digestion. As can be seen in Figure 2, the TPC remained statistically comparable before and after gastric digestion, while significantly increased after intestinal digestion. To the best of our knowledge, no data investigate the TPC content after simulated GID for the selected SWs.

The digested extracts were then analyzed for their capacity to inhibit angiotensin-converting enzyme (ACE). As reported in Table 7, all the analyzed algae have the potential to inhibit ACE with quantities ranging from 0.2 to 2.2 mg of DW.

## 3. Discussion

Edible algae represent an important food in Eastern countries, which discovered in antiquity the health advantages of consuming seaweeds. In this context, the use of marine algae dates back to the fourth and sixth centuries in China and Japan, respectively [36]. The three countries that consume seaweeds the most nowadays are Japan, China, and the Republic of Korea (FAO, 2003). Considering the nutrient profile, the benefits associated with the consumption of SWs are represented by the high content of fibers, proteins, and the quality of fats, rich in PUFAs. To date, VA and CL are considered infesting SWs in our territory and are treated as waste, with important disposal costs. We found that VA and CL, traditionally used as food in Eastern regions but not yet approved for human consumption in Europe, have the potential to be farmed as an important source of nutrients and nutraceutical compounds.

The nutrient composition is related to the species, territory and harvesting period [23], in this regard our data are in accord with those reported for PP by Dawczynski et al. (2007) [37]. Seasonal changes seem to be particularly important for nutrient composition [23]. As previously reported the total fat is negligible [24], accounting for less than 2% of the dry weight, while the protein content ranges from 8.10% DW in SJ to 16% DW in VA, 29% DW in CL, to 31% in PP. The total lipid amount found in SJ is 1.18, in perfect agreement with values reported in the period May–June (1.09–1.23). The protein content is related to the harvesting period being higher from February to May, accordingly, we found a value of 8.18%, comparable to that reported by Hwang (2014). Regarding PP, according to values determined, the fat content is usually comprised between 1.0 and 2.8%, while proteins are comprised between 30 and 33% [27]. CL has approximately 29% proteins, a value comparable with PP and others already determined in previous works, while VA has a protein content of approximately 16% DW [25,26], a value 2-fold lower than PP but 2-fold higher than SJ.

Regarding the amino acidic composition, all the SWs have a balanced amino acidic composition and contain all the essential amino acids. Amino acid composition influences food taste, we found an important concentration of Glu and Asp, which are responsible for the umami taste and sourness of SWs [38]. According to FAO and WHO recommended values (Table S2, Supporting Information), among unapproved SWs, VA has three limiting amino acids, Val, Lys and Leu, while exceeding the suggested score for all the remaining essential amino acids. CL has lower concentrations of His, Leu and Val. PP has a high concentration of all the essential amino acids, except His, in contrast, SJ fulfils the recommended values for all the amino acids. According to estimates, South Koreans consume 8.5 g/adult daily, Japanese consume 4 g/adult daily, and Chinese consume 5.2 g/adult daily [39]. Due to the high quantity of iodine and heavy metals, EFSA suggested to limit the consumption of unprocessed SWs, and a daily intake of a maximum of 4 g [40,41]. Considering this maximum quantity, the maximum daily apport of proteins ranges from 0.32 to 1.23 g of proteins for approved SWs, while VA and CL can apport 0.66 and 1.16 g of proteins, respectively.

Antioxidant compounds are beneficial to prevent injuries due to oxidative stress. As reported by Lanfer-Marquez et al., free radicals, particularly reactive oxygen species (ROS), initiate oxidative stress, which is a major factor in the pathophysiology of atherosclerosis, alcoholic liver cirrhosis, cancer, etc. [42]. SWs have a high content of hydrophilic antioxidant compounds. As already reported, SWs are rich in phlorotannins, essential to prevent oxidative stress and endowed with high antioxidant activity [43]. PP and CL are characterized by the higher content of polyphenols, flavonoids and proanthocyanidins, while VA and SJ have the lower content. Data are comparable to those reported by Farvin et al. [31], but higher than those reported by Stabili et al. [21]. The quantity of phenolic compounds can be modified by the solvent adopted for extraction, as well as using ultrasound or microwave-assisted procedure, for these reasons, the use of hydroalcoholic mixture coupled with ultrasound-assisted extraction is probably responsible for the higher phenolic recovery. Accordingly, Wang, Jónsdóttir, and Ólafsdóttir (2009) disclosed that polar organic solvents were superior to water for the extraction of polyphenolic from SWs [44]. The antioxidant activity calculated with ABTS and DPPH assays strongly correlates with the content of flavonoids and proanthocyanidins (Table S1, Supporting Information).

The lipophilic components of SWs were also analyzed, the extraction was performed using the Folch method, to prevent the alteration of metabolites due to the use of high temperatures. A macroscopic analysis of the main components has been performed using the NMR technique. As a result, the region between 0 and 5 ppm revealed the presence of fatty saturated and unsaturated fatty acids, sterols and triacylglycerols, while carotenoids and xanthophylls were found between 6.5 and 6.8 ppm. The magnification of the region between 8.5 and 11.5 revealed the presence of chlorophylls.

Lipophilic antioxidants chlorophyll, pheophytin and carotenoids were thus determined using spectrophotometric assays. CL was characterized by the highest content of antioxidant pigments. Chlorophylls play a pivotal role in algal photosynthesis and ensure the algal defense against excessive UV radiation. Among antioxidant compounds chlorophylls and carotenoids are of particular interest, as pigments for the pharmaceutical, cosmetic, and food industries (respectively E140 and E160). We found that CL has a total quantity of chlorophylls comparable to spinach, followed by PP and SJ with values major of green papric and VA with values comparable to that reported for apple peel [45]. The quantity of chlorophyll a is slightly below the content of chlorophyll b, probably due to the organic solvent used, which favors the extraction of the most lipophilic chlorophyll b. The market of organic pigments is actually valued of USD 4.4 billion, and is expected to grow at a CAGR of 6.5% in the next five years [46]. Considering that natural E140 pigment is commonly extracted from spinach, CL can represent an alternative source to recover chlorophyll pigment.

Through the action of organic acids, Mg^2+^ is removed from the center of the porphyrin ring of the chlorophylls to generate pheophytins. The content of pheophytins found without the addiction of MgCO_3_ ranges from 298.75 in VA to 4476.42 in CL, which represents the sum of the original content plus the amount formed during the extraction process.

Carotenoids are another class of lipophilic antioxidants with many uses in the pharmaceutical and food sectors. Carotenoids are contained in fruit and vegetable, but SWs represent an important source of these pigments. We found a total carotenoid content ranging from 1.92 to 60.62 µg/g DW, slightly below the ranges already reported after extraction with methanol [32,35]. Even in this case, the solvents used for the extraction can increase the extraction yields, Osorio et al. reported significantly higher yields in the carotenoid content after extraction of the *Porphyra* species with methanol instead of acetone, ethanol or DMF [35]. In addition, as already reported in different studies, sun-drying can reduce carotenoid content [47,48].

Fatty acids were identified by GC-MS and the individual fatty acid content quantified by GC coupled with a FID detector. Among the already approved SWs, SJ has the lowest content of SFAs, and the highest content of UFAs. The total quantity of fat is limited, accounting for less than 2% DW. The content of MUFAs in VA and CL is, respectively, 15% and 49% higher than PP, but the content of PUFAs is reduced by 27% and 38%. The n6/n3 ratio is below 1 for all the SWs, except PP; considering that a ratio below 10 is suggested to prevent cardiovascular diseases, both VA and CL are particularly suitable for controlled regimens. Considering the nutritional indexes associated with cardiovascular disease prevention, VA and CL have AI comparable to PP, in accordance with data already published for green SWs. TI is also comparable with values reported for green SWs, with a value 80% lower than green algae like *Ulva lactuga*, already included in the list of foods in Europe [49]. Taken together, the low quantity of fat coupled with an important content of UFAs and PUFAs makes algae particularly adapted for diet-controlled regimens, as demonstrated by nutritional indexes values.

Gastrointestinal digestion (GID) is a complex process characterized by enzymatic and mechanical conversion of food into bioavailable nutrients. Investigating the multi-stage digestion process of food in humans and animals is technically challenging and costly, for these reasons, in vitro GID models are useful to predict the real bioavailability of nutrients. We found that GID has important effects on TPC. In fact, even if before GD the TPC was slightly below the values reported in Table 3, due to the absence of methanol as extraction solvent, after gastric and intestinal digestion TPC increased, due to the hydrolysis of aglycons and phlorotannins, largely contained in algae. The two-way ANOVA found individual effects for SWs (df = 3, F = 2806, *p* < 0.0001) and digestion steps (df = 2, F = 332.3, *p* < 0.0001). A significant interaction was found between SWs and digestion steps (df = 6, F = 23.96, *p* < 0.0001). These results demonstrate that the nature of the solvent used for extraction influences TPC, as well as GID phases, highlighting that the TPC is often underestimated [50].

The metalloenzyme angiotensin-I-converting enzyme (ACE) is essential for the Renin-angiotensin system modulation of blood pressure [51,52]. As an exo-peptidase, it cleaves the C-terminal dipeptide of angiotensin I to yield the octapeptide angiotensin II, a strong vasoconstrictor. As reported for many years now, angioedema, dysgeusia, cough, hyperkalemia, renal failure, fetal malformations, and skin rashes are among the many side effects of ACE synthetic inhibitors [53]. Different studies demonstrate that many compounds with ACE inhibitory activity are contained in foods, among them polyphenols and phlorotannin contained in seaweeds. Previous reports highlight that gastrointestinal digestion generates peptides with ACE inhibitory activity in the human body. These peptides can be derived from many different animal and plant proteins like fish, insects, milk or soy [54,55]. Other authors have already reported on the remarkable ACE inhibitory effects of enzymatic hydrolysates from various macroalgae, particularly from brown and red algal species [56,57,58]. Among SWs, we found that PP extract has the better half maximal inhibitory activity (IC_50_) value of the series, followed by SJ, CL and VA. Other red algae exhibit potent ACE inhibitory activity, due to phlorotannins, lipids and peptides [59,60]. Interestingly, the activity of brown SWs has been also screened before GID, with inhibitory percentages ranging from 16 to 60% for *Porphyra* species [60,61]. After GID, the potency increases, with values closer to those found by us, [62]. Similar results have been reported for brown and green SWs, in particular the aqueous extraction increased the inhibitory activity of CL with respect to hydroalcoholic mixtures [22].

Even if CL and VA are not yet approved in Europe, this study demonstrates that are a source of high-quality nutrients and nutraceuticals with antioxidant and ACE inhibitory activity. As strongly promoted by the European Green Deal, the Farm to Fork Strategy and the Sustainable Blue Economy Communication, additional efforts are necessary to promote the use of algae as food and feed [4,5]. The positive opinion of EFSA is strictly required to commercialize SWs like CL and VA as novel foods in Europe. Even if the long authorization process and the costs associated with the preparation of data discourage filling novel applications, the possible benefits are remarkable. On the one hand, the commercialization of CL and VA can give important economic revenues and environmental benefits; on the other hand, CL and VA are a complete source of high-quality nutrients and antioxidant compounds. Even if robust data are necessary to limit the risks associated with novel foods, novel strategies should be investigated to promote novel applications. Affordable strategies can be represented by the reduction in the timelines for authorization or the introduction of simplified applications for foods already consumed for a long time in other territories. In fact, timelines for applications are often too long for SMEs, with reduced financial capabilities.

## 4. Materials and Methods

### 4.1. Materials

The solvents and reagents used (analytical grade) were obtained from Sigma-Aldrich S.r.l. (Milan, Italy) and used without additional purification. Milli-Q quality water (Millipore, Milford, MA, USA) was used. *Valonia aegagrophila* (*VA*) and *Chaetomorpha linum* (CL) were harvested in Tuscany in May, as a kind gift of “Orbetello pesca lagunare” (Orbetello, Italy). Nori (*Porphyra purpurea*, PP) and Kombu (*Saccharina japonica* SJ) were purchased in a local market. Seaweeds (SWs) were air-dried for 48 h, and the moisture content was reduced to 10–12% *w*/*w* by drying under vacuum at 20 °C for 24 h. Dried seaweeds were finely pulverized using a Bosch food processor TSM6A013B, (Bosh, Gerlingen-Schillerhoehe, Germany). After being weighed, the samples were kept at −20 °C until analysis.

### 4.2. Preparation of Extracts from Seaweeds

Main hydrophilic components were extracted starting from 1 g of dried SWs. SWs were homogenized for 10 min with an IKA Labortechnik T25 basic (IKA Werke GmbH & Co., Staufen, Germany) in a mixture of MeOH/H_2_O 1/1 (20 mL) and subjected to extraction in an ultrasonic bath for 10 min at room temperature. Supernatant and pellet were separated after centrifugation at 74,689× *g* for 5 min (Eppendorf 5430, Eppendorf, Germany), and the pellet was then extracted again using the same method. A PTFE BUCHI mod. A V-100 pump (Buchi, Flawil, Switzerland) was used to remove the solvent at reduced pressure (10 mbar). The total phenol content, antioxidant activity, and radical scavenging activity of the hydrophilic extract were assessed. The same procedure has been adopted to extract pigments, starting from 50 mg of dried SWs, using 2 mL of acetone.

The Folch method was used to extract lipids, starting from 5 g of dried SWs [63]. After being weighed to determine the fat percentage, the residue was used for FAs analysis.

### 4.3. Analysis of Proximate Composition

Analysis of moisture was carried out using the oven drying technique. Approximately 1 g of each sample was weighed wet, and the moisture content was calculated by subtracting the water content over the course of 14–16 h at 105 °C.

Protein content has been determined using the Kjeldahl method. The assay consists of digestion, distillation and titration of the sample (1.0 g). The sample was digested in 20 mL of sulfuric acid at 300 °C for 60 min. After this time, H_2_O and NaOH 40% (*w*/*w*) were added (50 mL) and the solution was distilled and titrated and 25 mL of distilled and titrated with HCl.

The crude protein content was determined using a conversion factor of 6.25.

Energy values were calculated by using the following factors: 9.02 kcal/g for fats × 1, 3.87 kcal/g for carbohydrates and ×4.27 kcal/g for proteins.

### 4.4. Determination of Fatty Acids

FAs (10 mg) have been converted into their methyl esters by reacting with 1 mL of BF_3_ methanolic solution, for 1 h at 100 °C [33].

The GC-FID analyses were performed using a GC-FID (Perkin Elmer Clarus 500 GC, Waltham, MA, USA) and a split/spitless injector as previously reported [64]. By examining the FA areas and their percentage for each resolved peak, the FAs were quantified.

### 4.5. Determination of Amino Acids

Amino acids (AA) were obtained after 24 h acidic hydrolysis at 110 °C. AA were quantified by HPLC with an Agilent 1260 Infinity instrument (Agilent Technologies, Palo Alto, CA, USA) using the Fmoc pre-derivatization procedure and the chromatographic conditions already reported [65]. Separation was obtained with a LC-18 column (Gemini NxC18, 4.6 × 250 mm, 5 μm), using acetonitrile as eluent B and 50 mM acetate buffer (pH 4.2) as eluent A. The column was run at 1.0 mL/min at 25 °C.

The following linear gradient elution conditions (min/A%) were used to separate the amino acids: 0/72, 3/72, 27/55, 32/0, 37/0, 39/72, and 47/72.

### 4.6. Quantification of Antioxidant Compounds

The Folin–Ciocalteu method with gallic acid as a standard was used to determine total polyphenol content (TPC) as previously reported [66]. In detail, 100 µL of each sample, properly diluted, was combined with 200 µL of the Folin–Ciocalteu reagent and vortexed. Then, 1.7 mL of water and 1 mL of a 10% *w*/*v* sodium carbonate aqueous solution were added. After 30 min of incubation, the absorbance was recorded at 765 nm (UV/Visible Lambda 2 spectrophotometer, Perkin-Elmer, Norwalk, CT, USA). The TPC was indicated as mg of gallic acid equivalent per gram of dry matter (mg GAE/g DW).

Total flavonoid content (TFC) has been determined as previously reported [67], using quercetin as a standard. In detail, 60 µL of 10% AlCl_3_ in EtOH and 60 µL of 1 M CH_3_COONa were combined with 300 µL of the opportune extract. 900 µL of EtOH and 1680 µL of water were added, and the mixture was vortexed. After 40 min of incubation, the absorbance was recorded at 415 nm. The TFC was indicated as mg of quercetin equivalent per gram of dry matter (mg QE/g DW). Total Proanthocyanidin Content (PATC) has been determined as previously reported [67], using catechin as a standard. 200 µL of each sample, properly diluted, were combined with 2800 µL of vanillin HCl reagent. The mixture was vortexed and after 20 min of incubation, the absorbance was recorded at 500 nm. The PATC was indicated as mg of catechin equivalent per gram of dry matter (mg CE/g DW).

1,1-diphenyl-2-picrylhydrazyl (DPPH) assay was used to determine radical scavenging activity as reported [68], using Trolox as a reference. 1.0 mL of 0.2 mM DPPH solution was combined with 100 µL of extract and 1.9 mL of MeOH. The mixture was vortexed, and allowed to react in dark static conditions. After 30 min, the absorbance was recorded at 517 nm The radical scavenging activity was indicated as TEAC (µmol of Trolox equivalent per gram of dry matter).

2,2′-azino-bis(3-ethylbenzothiazoline-6-sulfonic acid) (ABTS) radical cation decolorization assay was adopted to determine the antioxidant activity as already published [68], by employing Trolox as a reference. A volume of 100 µL of the opportune hydrophilic extract was added to 1.5 mL of ABTS solution and EtOH was added to reach a final volume of 3 mL. The mixture was vortexed and after 10 min, the absorbance was recorded at 751 nm. The antioxidant activity was indicated as TEAC (µmol of Trolox equivalent per gram of dry matter).

### 4.7. Gastrointestinal Digestion

Gastrointestinal digestion (GID) has been determined in vitro as previously published in [55] with some modifications. An amount of 500 mg of dried SWs was homogenized for 5 min in 5 mL of TRIS/HCl buffer 50 mM pH 7.4, using an IKA Labortechnik T25 basic (IKA Werke GmbH & Co., Staufen, Germany). After this time, the pH was adjusted to 2 by adding HCl 1M, and pepsin was added (enzyme/substrate 1:250 *w*/*w*). The mixture was incubated for 2 h at 37 °C. The extract has been used to determine the effect of gastric digestion (GD). The pH was then increased to 6.5 with NaOH 1M, and a mixture of trypsin and α-chymotrypsin (1:1) was added (enzyme/substrate 1:250 *w*/*w*). The mixture was incubated for 2 h at 37 °C. The samples were centrifuged at 74,689× *g* for 10 min, the supernatant was collected and filtered on 3 kDa filters (Amicon Ultra Millipore Sigma, Burlington, MA, USA) and kept at −20 °C until use.

### 4.8. ACE inhibitory Activity Assay

ACE inhibitory activity was detected in GID extracts as previously reported [55]. Scalar concentrations of each sample, 25 μL of ACE 0.1 mU/mL (ACE enzyme from rabbit lung purchased from Sigma-Aldrich, St. Louis, MI, USA) and 40 μL of HHL 6.5 mM, were incubated for 30 min at 37 °C. The enzyme reaction was terminated by adding 85 μL of HCl 1 M. Blank sample was obtained replacing sample solution with borate buffer. All samples were filtered before RP-HPLC analysis.

### 4.9. LC Determination of HA

ACE inhibitory activity was detected by quantifying hippuric acid by liquid chromatography, with an Agilent 1260 Infinity instrument (Agilent Technologies, Palo Alto, CA, USA) using the method previously published [55]. Separation was obtained with a LC-18 column (Phenomenex Ultracarb, Torrance, CA, USA, 4.6 × 150 mm, 5 μm), using an isocratic mixture of ACN (25%) and H_2_O containing 0.05% formic acid (75%) at a flow rate of 0.8 mL/min.

ACE inhibitory activity was determined using the equation:

(1) $$\mathrm{ACE}\;\mathrm{inhibition}\;(\%)=(1-\frac{A\;inhibitor}{A\;blank})\times 100$$

*A_inhibitor_* is the peak area of HA, while *A_blank_* is the peak area of HA in the blank sample. The ACE inhibition was reported as half maximal inhibitory concentration (IC_50_).

### 4.10. NMR Analysis

Lipophilic extracts (20 mg) were solubilized in 0.7 mL of CDCl_3_, containing 0.03% (*v*/*v*) tetramethylsylane (Sigma Aldrich, Darmstadt, Germany). Monodimensional ^1^HNMR were recorded at T = 298 K, using a Bruker Advance DPX 400 MHz spectrometer (Bruker Biospin, Billerica, MA, Germany). ^1^H NMR of lipophilic extracts were acquired with a single pulse experiment, with a 90° excitation pulse, 4 dummy scans, 3 s relaxation delay, and 16 scans. 1H NMR spectra were assigned by comparison with the published data [21,69] and Chenomx NMR suite v 11 (Chenomx Inc., Edmonton, AB, Canada)

### 4.11. Quantification of Lipid Quality Indices

The subsequent formulas were adopted to calculate indices of thrombogenicity (IT) and atherogenicity (*AI*):

(2) $$T=\frac{\left(C14:0)+(C16:0)+(C18:0\right)}{\left(0.5\times \sum MUFA\right)+\left(0.5\times \sum n6\;PUFA\right)+\left(3\times \sum n3\;PUFA\right)+\left(\sum n3/\sum n6\right)}$$

(3) $$AI=\frac{\left(C12:0)+(4\times C14:0)+(C16:0\right)}{\left(PUFAs(\sum n6\;and\;\sum n3)+MUFA\right)}$$

### 4.12. Statistical Analysis

Experiments were replicated three times. All the experimental results were analyzed using GraphPad Prism 8.2 (GraphPad Software, La Jolla, CA, USA) and were reported as the mean plus standard deviations (SD). The data’s homoscedasticity was confirmed with the Brown–Forsythe test. The Shapiro–Wilk test was used to confirm the data’s normality. Nutrients, antioxidants, lipids, and amino acids were analyzed using one-way analysis of variance (ANOVA). To compare means, Tukey’s post hoc test was used. Only data with *p* ≤ 0.05 were considered significant. Two-way ANOVA with different gastrointestinal phases of digestion and SWs as fixed factors were used to analyze the total polyphenol content before, during and after digestion. Pearson correlation analysis was assayed between antioxidant activity (ABTS and DPPH) and antioxidant contents (polyphenols, flavonoids, and proanthocyanidins).

## 5. Conclusions

Oceans and algae represent a valuable and underused resource. Herein, two infesting SWs have been analyzed for their possible use as food or nutraceuticals. In fact, even if they are an ingredient in many Asian preparations, they represent a waste in Italy, with important disposal costs.

Herein, we demonstrate that VA and CL are a complete source of proteins, containing all the essential amino acids. The fat content is negligible, accounting for less than 2% DW, but it is of high quality due to the presence of UFAs. VA and CL are also a source of antioxidant compounds, including pigments with high economic value. Gastrointestinal digestion has been evaluated, indicating that the quantity of polyphenols increased due to intestinal enzymes. Most importantly, both SWs have ACE inhibitory activity, which is important to reduce hypertension and categorize SWs as a functional food. Even if the in vitro data are compelling, future studies are necessary to prove the antihypertensive activity of CL and VA in in vivo preclinical models.

## Figures and Tables

**Table 1:** Analysis of nutrients in seaweeds *: residual water content, proteins, and fats.

	Residual Water (g/100 g DW)	Fats (g/100 g DW)	Proteins (g/100 g DW)
	Mean ± SD	Mean ± SD	Mean ± SD
VA	8.50 ± 0.71 ^a^	1.46 ± 0.06 ^b^	16.61 ± 0.73 ^b^
CL	7.50 ± 0.69 ^a^	1.62 ± 0.14 ^c^	28.9 ± 0.81 ^c^
PP	12.50 ± 0.80 ^b^	1.09 ± 0.15 ^a^	30.72 ± 0.38 ^c^
SJ	6.85 ± 0.57 ^a^	1.18 ± 0.17 ^a^	8.10 ± 0.51 ^a^

* Results represent the mean ± SD of three experiments. *Valonia aegagrophila* (VA), *Chaetomorpha Linum* (CL) Nor*i* (*Porphyra purpurea*, PP) and Kombu (*Saccharina japonica* SJ). Values are expressed as g per 100 g of dry weight. ^a–c^ Different superscript letters indicate a significant difference among the means in each column; *p* < 0.05 by post hoc Tukey’s test.

**Table 2:** Amino acid composition of seaweeds reported in means ± SD in seaweeds (g/100 g DW).

		VA ±SD	CL ±SD	PP ±SD	SJ ±SD
Essential					
	His	0.091 ± 0.003	0.100 ± 0.008	0.399 ± 0.025	0.200 ± 0.021
	Thr	0.755 ± 0.045 ^b^	1.039 ± 0.075 ^b^	1.467 ± 0.135 ^c^	0.399 ± 0.015 ^a^
	Val	0.026 ± 0.005 ^a^	0.225 ± 0.019 ^a^	1.998 ± 0.251 ^c^	0.890 ± 0.051 ^b^
	Met	0.203 ± 0.011	0.515 ± 0.061	0.453 ± 0.036	0.251 ± 0.010
	Lys	0.074 ± 0.003 ^a^	1.746 ± 0.154 ^c^	1.708 ± 0.154 ^c^	0.700 ± 0.051 ^b^
	Ile	2.866 ± 0.198 ^c^	3.691 ± 0.410 ^d^	1.279 ± 0.129 ^b^	0.410 ± 0.028 ^a^
	Leu	0.710 ± 0.056 ^a^	0.795 ± 0.104 ^a^	1.851 ± 0.157 ^b^	0.648 ± 0.051 ^a^
	Phe	0.173 ± 0.008 ^a^	0.236 ± 0.019 ^a^	1.271 ± 0.098 ^c^	0.398 ± 0.040 ^b^
Total EAA		4.898 ± 0.329 ^a^	8.348 ± 0.850 ^b^	10.426 ± 0.985 ^c^	3.896 ± 0.267 ^a^
Non-Essential	Asp	1.366 ± 0.098 ^a^	2.446 ± 0.261 ^ab^	2.969 ± 0.310 ^b^	0.591 ± 0.041 ^a^
	Ser	0.483 ± 0.029 ^a^	0.662 ± 0.0582 ^a^	2.239 ± 0.239 ^b^	0.360 ± 0.025 ^a^
	Glu	2.504 ± 0.157 ^b^	5.305 ± 0.481 ^c^	3.045 ± 0.410 ^b^	0.750 ± 0.057 ^a^
	Gly	2.433 ± 0.142 ^b^	3.689 ± 0.256 ^b^	1.965 ± 0.152 ^b^	0.565 ± 0.032 ^a^
	Arg	0.023 ± 0.001 ^a^	1.498 ± 0.151 ^b^	1.106 ± 0.109 ^b^	0.297 ± 0.018 ^b^
	Ala	2.303 ± 0.135 ^b^	3.694 ± 0.410 ^c^	3.066 ± 0.295 ^bc^	0.652 ± 0.041 ^a^
	Pro	0.264 ± 0.041 ^a^	0.357 ± 0.027 ^a^	1.407 ± 0.162 ^b^	0.114 ± 0.053 ^a^
	Cys	0.561 ± 0.037	0.071 ± 0.006	0.305 ± 0.028	0.294 ± 0.018
	Tyr	0.382 ± 0.021 ^a^	0.669 ± 0.081 ^a^	1.576 ± 0.131 ^b^	0.285 ± 0.021 ^a^
Total Non-EAA		10.318 ±0.661 ^b^	18.390 ±1.731 ^d^	17.678 ±1.836 ^c^	3.908 ± 0.306 ^a^
Total AA		15.216 ±0.990 ^b^	26.739 ±2.581 ^c^	28.104 ±2.821 ^d^	7.804 ± 0.573 ^a^

^a–d^ Values in a row without a common superscript are significantly different (*p* < 0.05).

**Table 3:** Quantification of phenolic compounds in seaweeds *.

	Total Polyphenols (mg GA/g DW)	Flavonoids (mg QE/g DW)	Proanthocyanidin (mg CE/g DW)
	Mean ± SD	Mean ± SD	Mean ± SD
VA	1.03 ± 0.09 ^a^	1.02 ± 0.07 ^a^	0.57± 0.02 ^a^
CL	2.36 ± 0.02 ^b^	2.69 ± 0.06 ^b^	0.66 ± 0.01 ^a^
PP	4.82 ± 0.30 ^c^	29.67 ± 1.15 ^c^	5.26 ± 0.37 ^b^
SJ	1.10 ± 0.03 ^a^	1.11 ± 0.15 ^a^	0.52 ± 0.00 ^a^

* Results represent the mean ± SD of three experiments. *Valonia aegagrophila* (VA), *Chaetomorpha linum* (CL), Nor*i* (*Porphyra purpurea,* PP) and Kombu (*Saccharina japonica* SJ). Flavonoids are expressed as mg of quercetin equivalent (QE) per gram of dry weight; condensed tannins are expressed as mg of catechin equivalent (CE) per gram of dry weight. ^a–c^ Different superscript letters indicate a significant difference among the means in each column, *p* < 0.05 by post hoc Tukey’s test.

**Table 4:** Analysis of antioxidant (ABTS) and radical scavenging (DPPH) activities of seaweeds expressed as the mean ± SD *.

	TAC (µmol Trolox/g DW)	
	ABTS	DPPH
VA	5.76 ± 0.01 ^a^	445.49 ± 12.24 ^c^
CL	5.69 ± 0.23 ^a^	350.32 ± 24.04 ^b^
PP	14.44 ± 1.35 ^b^	1302.48 ± 20.53 ^d^
SJ	4.17 ± 0.33 ^a^	17.82 ± 1.95 ^a^

* Results represent the mean ± SD of three experiments. *Valonia aegagrophila* (VA), *Chaetomorpha Linum* (CL), Nor*i* (*Porphyra purpurea*, PP) and Kombu (*Saccharina japonica* SJ). ABTS and DPPH are expressed as µmol of Trolox equivalent per gram of dried weight. ^a–d^ Different superscript letters indicate a significant difference among the means in each column, *p* < 0.05 by post hoc Tukey’s test.

**Table 5:** Total fat and FA composition of the standard diet and nursey wastes expressed as the mean ± SD *.

	VA	SD	CL	SD	PP	SD	SJ	SD
C10:0	0.07	0.02	0.37	0.04	0.09	0.00	0.01	0.00
C12:0	0.15	0.02 ^a^	3.11	0.34 ^b^	0.07	0.00 ^a^	0.06	0.00 ^a^
C13:0	0.02	0.00	0.13	0.01	0.18	0.02	0.00	0.00
C14:0	7.04	0.53 ^b^	7.06	0.30 ^b^	4.66	1.28 ^a^	7.29	0.09 ^b^
C15:0	0.54	0.03	0.80	0.22	0.54	0.01	0.00	0.00
C16:0	45.74	0.50 ^b^	40.34	0.70 ^b^	45.66	4.13 ^b^	24.82	1.07 ^a^
C17:0	0.36	0.03	0.00	0.00	0.49	0.14	0.00	0.00
C18:0	1.99	0.86	1.42	0.02	1.24	0.17	1.64	0.21
C20:0	0.61	0.06	0.71	0.32	1.01	0.10	1.82	0.34
C22:0	0.80	0.16	0.27	0.03	0.00	0.00	0.19	0.03
C24:0	1.01	0.03	0.88	0.06	0.16	0.03	0.00	0.00
Σ SFA	58.25	0.92 ^c^	54.73	1.41 ^b^	54.01	5.43 ^b^	35.83	1.02 ^a^
C16:1 9	6.12	0.23 ^b^	8.00	0.52 ^c^	6.05	0.24 ^b^	3.71	0.00 ^a^
C16:1 11	4.70	0.24 ^a^	11.25	0.62 ^c^	6.37	1.04 ^a^	5.42	0.24 ^a^
C18:1 9	1.60	0.04 ^a^	5.79	0.78 ^b^	3.70	0.21 ^ab^	30.78	0.17 ^c^
C18:1 11	7.99	1.23 ^c^	3.58	0.18 ^b^	0.73	0.29 ^a^	0.00	0.00
C20:1 11	1.77	0.21 ^a^	0.11	0.04 ^a^	2.40	0.11 ^a^	0.00	0.00
Σ MUFA	22.17	0.92 ^b^	28.73	1.10 ^c^	19.25	0.67 ^a^	39.92	0.07 ^d^
C16: 3 4 7 10	2.69	0.52 ^a^	5.94	0.25 ^b^	4.68	0.57 ^ab^	3.67	1.27 ^a^
C16: 2 9 11	0.00	0.00	1.65	0.27	0.78	0.79	1.03	0.22
C18:2 9 12	6.26	0.70 ^ab^	7.79	0.90 ^b^	4.61	0.04 ^a^	6.00	0.19 ^ab^
C18 :3 9,12,15	8.21	0.10 ^b^	0.00	0.00	0.43	0.05 ^a^	0.31	0.05 ^a^
C18: 5 5 8 11 14 17	0.00	0.00	0.00	0.00	0.00	0.00	5.63	1.49
C20:4 5 8 11 14	0.53	0.11 ^a^	0.38	0.03 ^a^	2.20	0.12 ^b^	5.06	0.16 ^b^
C20: 5 5 8 11 14 17	1.29	0.10 ^a^	0.78	0.07 ^a^	14.04	0.40 ^b^	2.55	0.06 ^a^
C22:6	0.60	0.04	0.61	0.04	0.00	0.00	0.00	0.00
Σ PUFA	19.58	1.37 ^b^	16.54	0.82 ^a^	26.74	0.21 ^c^	24.25	2.87 ^c^
Σ n6	6.80	0.51 ^b^	1.39	0.03 ^a^	14.46	0.35 ^c^	2.86	0.01 ^a^
Σ n3	10.05	0.03 ^d^	8.39	1.08 ^c^	0.86 ^a^	0.01	4.96	0.18 ^b^
Σ n6/Σ n3	0.68	0.08 ^a^	0.17	0.05 ^a^	16.77	0.01 ^b^	0.58	0.14 ^a^
AI	1.78	0.02 ^b^	1.58	0.12 ^b^	1.40	0.19 ^b^	0.84	0.06 ^a^
TI	1.23	0.01 ^b^	1.21	0.13 ^b^	2.65	0.11 ^c^	0.93	0.05 ^a^

* Results represent the mean ± SD of three experiments. *Valonia aegagrophila* (VA), *Chaetomorpha Linum* (CL) Nor*i* (*Porphyra purpurea*, PP) and Kombu (*Saccharina japonica SJ*). Atherogenicity index (AI), thrombogenicity index (TI). Nd: not detected. ^a–d^ Different superscript letters indicate a significant difference among the means in each column, *p* < 0.05 by post hoc Tukey’s test.

**Table 6:** Quantification of lipophilic antioxidant compounds in seaweeds *.

	Chlorophyll-α (µg/g DW)	Chlorophyll-β (µg/g DW)	Total Chlorophylls (µg/g DW)	Total Pheophytins (µg/g DW)	Total Carotenoids (µg/g DW)
VA	27.39 ± 1.71 ^a^	37.05 ± 1.01 ^a^	64.44 ± 1.52 ^a^	298.75 ± 19.23 ^a^	3.78 ± 0.20 ^a^
CL	409.84 ± 15.01 ^c^	574.74 ± 3.19 ^c^	984.58 ± 9.27 ^c^	4476.42 ± 45.53 ^b^	60.62 ± 2.01 ^c^
PP	60.22 ± 4.02 ^b^	75.95 ± 4.03 ^b^	136.17 ± 4.03 ^b^	614.87 ± 29.25 ^a^	1.92 ± 0.28 ^a^
SJ	73.39 ± 5.21 ^b^	82.66 ± 5.04 ^b^	156.05 ± 5.13 ^b^	686.09 ± 34.02 ^a^	22.06 ± 1.91 ^b^

* Results represent the mean ± SD of three experiments. *Valonia aegagrophila* (VA), *Chaetomorpha Linum* (CL) Nori (*Porphyra purpurea*, PP) and Kombu (*Saccharina japonica* SJ). ^a–c^ Different superscript letters indicate a significant difference among the means in each column, *p* < 0.05 by post hoc Tukey’s test.

**Table 7:** Analysis of angiotensin-converting enzyme (ACE) inhibitory activity after gastrointestinal digestion (GID). Values are expressed as the mean IC_50_ ± SD *.

	IC_50_ (mg DW)
VA	5.71 ± 1.15 ^c^
CL	3.02 ± 0.49 ^b^
PP	0.57 ± 0.03 ^a^
SJ	2.85 ± 0.07 ^b^

* Results represent the mean ± SD of three experiments. *Valonia aegagrophila* (VA), *Chaetomorpha Linum* (CL) Nor*i* (*Porphyra purpurea*, PP) and Kombu (*Saccharina japonica* SJ). IC_50_: Quantity of dry SWs needed to inhibit 50% of the ACE activity. ^a–c^ Different superscript letters indicate a significant difference among the means in each column, *p* < 0.05 by post hoc Tukey’s test.

## Data Availability

Data are available on request.

## Supplementary Materials

The following supporting information can be downloaded at: https://www.mdpi.com/article/10.3390/ijms25147588/s1.
