# Supplementary material for: Infesting Seaweeds as a Novel Functional Food: Analysis of Nutrients, Antioxidants and ACE Inhibitory Effects
Source: Int J Mol Sci. 2024 Jul 10;25(14):7588. doi: 10.3390/ijms25147588 (PMC11277057; doi:10.3390/ijms25147588)
Supplement: Supplementary file 1 [file ijms-25-07588-s001.zip › ijms-3082666-supplementary.pdf]

Contents

Table S1. Pearson's correlation for antioxidant activities (ABTS and DPPH) versus antioxidant content (Flavonoids, Proanthocyanidins and Polyphenols). 3

Figure S1. Magnification of *Chaetomorpha linum* spectrum. 4

Table S2. Aminoacidic composition of *Valonia aegagrophila* (VA), *Chaetomorpha Linum* (CL) Nori (*Porphyra purpurea*, PP) and Kombu (*Saccharina japonica* SJ) compared with FAO/WHO/UNU suggested amino acid requirements in mg/g of protein. 5

Table S1. Pearson's correlation for antioxidant activities (ABTS and DPPH) versus antioxidant content (Flavonoids, Proanthocyanidins and Polyphenols). Values in bold are significant.

	ABTS		DPPH	
	r	p	r	p
Flavonoids	**0.9898**	0.0102	**0.9455**	0.0545
Proanthocyanidins	**0.9902**	0.0098	**0.9471**	0.0529
Polyphenols	0.8812	0.1188	0.8541	0.1459
DPPH	**0.9823**	0.0177	**---**	---

R = correlation coefficient; p= p value

Figure S1. Magnification of *Chaetomorpha linum* 1H NMR spectrum.

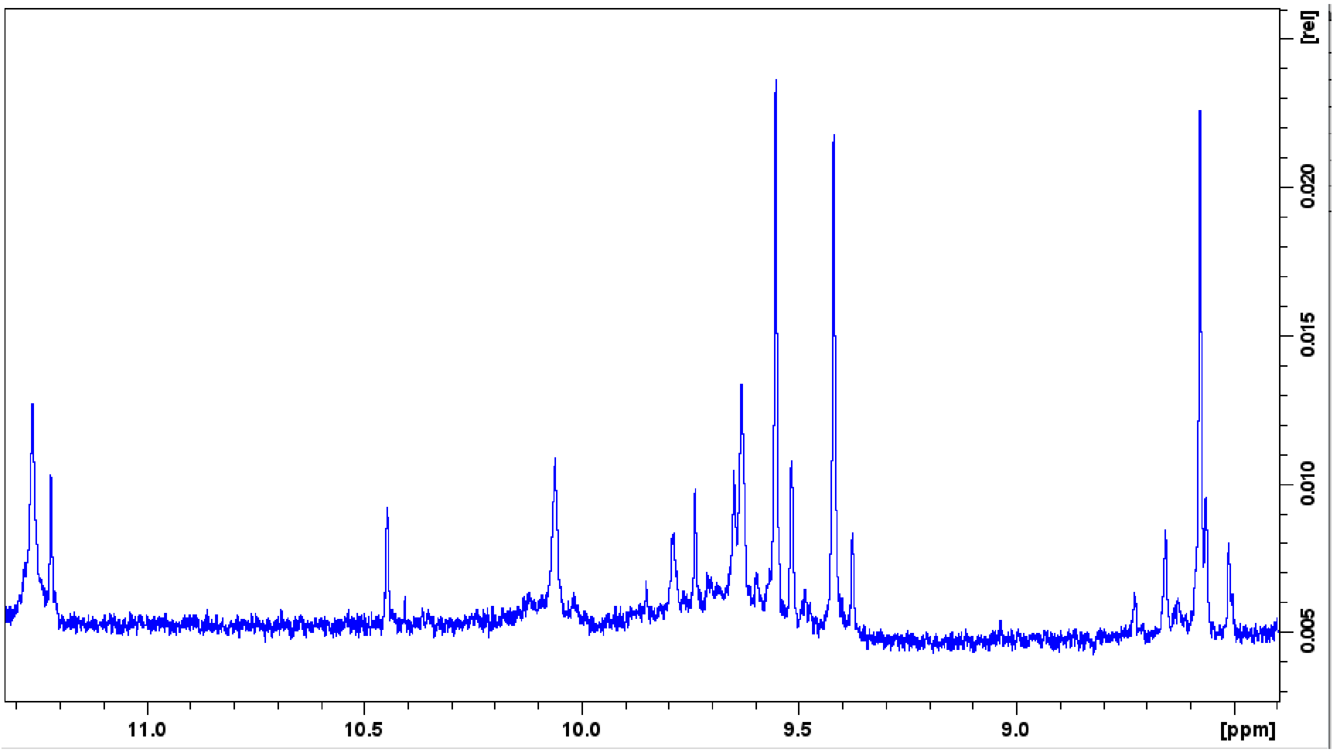

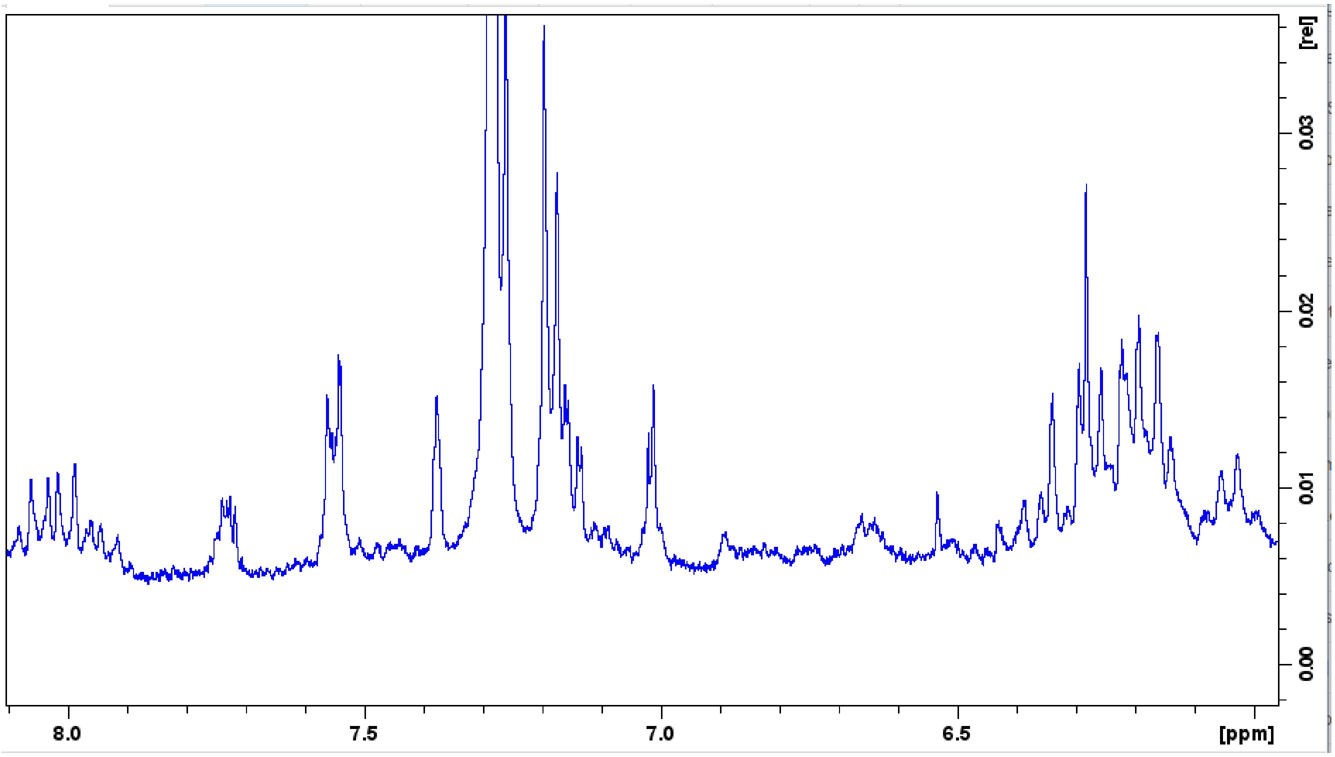

Table S2. Aminoacidic composition of *Valonia aegagrophila* (VA), *Chaetomorpha Linum* (CL) Nori (*Porphyra purpurea*, PP) and Kombu (*Saccharina japonica* SJ) compared with FAO/WHO/UNU suggested amino acid requirements in mg/g of protein.

AA	VA	CL	PP	SJ	FAO/ WHO/ UNU reference protein
**His**	5.5	3.5	13.0	24.7	19
**Thr**	45.5	35.95	47.75	49.3	34
**Val**	1.6	7.8	65.0	109.9	35
**Met+Cys**	46.0	20.3	24.7	67.3	25
**Lys**	4.5	60.4	55.6	86.4	58
**Ile**	172.6	127.7	41.6	50.6	28
**Leu**	42.7	27.5	60.3	80.0	66
**Phe + Tyr**	33.4	31.3	92.7	84.3	63
**Total EAA**	294.9	288.9	339.4	481.0	328
**Met**	12.2	17.8	14.7	31.0	
**Cys**	33.8	2.5	9.9	36.3	
**Phe**	10.4	8.2	41.4	49.1	
**Tyr**	23.0	23.1	51.3	35.1	
